# Evaluating Food Intake of Post-Acute Myocardial Infarction Patients According to a European Guideline and Mediterranean Diet Score: DICA-NUTS Substudy

**DOI:** 10.3390/life15071051

**Published:** 2025-06-30

**Authors:** Rodrigo Damasceno de Oliveira, Lívia Costa de Oliveira, Marcio Santos Prazeres, Tais Saint Martin Fonseca, Aline Marcadenti, Angela Cristine Bersch-Ferreira, Rachel Helena Vieira Machado, Elisa Maia dos Santos, Annie Seixas Bello Moreira, Grazielle Vilas Bôas Huguenin

**Affiliations:** 1 Department of Education and Research, National Institute of Cardiology, Rio de Janeiro 20231-050, RJ, Brazil; damascen8@gmail.com (R.D.d.O.); elisamaia80@gmail.com (E.M.d.S.); 2 José Alencar Gomes da Silva National Cancer Institute—INCA, Rio de Janeiro 22780-160, RJ, Brazil; lillycostaoliveira@gmail.com; 3 Department of Nutrition, Salgado de Oliveira University, São Gonçalo 24456-570, RJ, Brazil; marcioprazeres.msp@gmail.com; 4 Department of Nutrition, Federal University of the State of Rio de Janeiro, Rio de Janeiro 20559-900, RJ, Brazil; taissaint@gmail.com; 5 Hcor Research Institute, Sao Paulo 04004-030, SP, Brazil; 6 Institute of Cardiology, University Foundation of Cardiology, Porto Alegre 90620-000, RS, Brazil; 7 School of Public Health, University of São Paulo, Sao Paulo 05508-220, SP, Brazil; 8 Applied Nutrition Department, State University of Rio de Janeiro, Rio de Janeiro 20550-013, RJ, Brazil; anniebello@gmail.com; 9 Nutrition and Dietetics Department, Federal Fluminense University, Niteroi 24210-200, RJ, Brazil

**Keywords:** Mediterranean diet, acute myocardial infarction, Mediterranean diet score, eating

## Abstract

Contextualization: Lifestyle changes, such as smoking cessation, physical activity, and healthy eating, are essential for the treatment and prevention of cardiovascular diseases. The 2021 update of the European Society of Cardiology (ESC) guidelines highlights the need to investigate the adherence to the Mediterranean diet in patients who have suffered acute myocardial infarction (AMI). Objective: The aim of this study was to investigate the adherence to the Mediterranean diet and dietary 2021 ESC guidelines of individuals who have suffered AMI and to evaluate the sociodemographic and lifestyle factors associated with the adherence. Methods: A cross-sectional study was conducted using baseline data from participants prior to enrollment in the DICA-NUTS multicenter clinical trial. The dietary intake was assessed using a food frequency questionnaire and 24 h dietary recall. The nutrient intake was compared with the nutritional recommendations of the 2021 ESC guidelines, and the adherence to the Mediterranean diet was analyzed using a Mediterranean diet score. Furthermore, the analyses of the factors associated with the Mediterranean diet score and 2021 ESC were performed. Analyses were conducted using Stata Data Analysis and Statistical Software version 15.0. Values were considered statistically significant when the *p*-value < 0.05. Results: Among 488 participants aged ≥ 40 years, moderate adherence to the Mediterranean diet was observed obtaining an average of nine points, with a low intake of vegetables, fish, and cereals. The protein intake was adequate with 18% (interquartile range [IQR] 15.0–23.1) of the total energy value, and the saturated fat intake was high with 9.7% (IQR 7.3–12.7) of the total energy value, while the carbohydrate and total fat intake was adequate. According to the 2021 ESC guidelines, the fiber intake was low with more than 79% of the sample consuming less than 30 g per day. The multivariate analysis using the 2021 ESC dietary recommendations showed that older age (≥60 years) [odds ratio (OR) = 1.63; 95% confidential interval (CI) = 1.44–1.91], never smoking (OR = 1.34; 95% CI = 1.17–1.65), and higher education (OR = 1.37; 95% CI = 1.17–1.77) were correlated with an increased fruit and vegetable intake. Non-white, never-smokers, and former smokers were more likely to consume fish. The alcohol scores were higher in older age participants and women. The dairy scores increased with older age, while the cereal scores decreased. No significant association was found for legumes. Conclusions: According to the scores used, the diet of these individuals moderately reflects the Mediterranean characteristics. Analyzing the 2021 ESC dietary recommendations, the studied population was inadequate. This study found different factors associated with an adequate food intake in post-AMI patients. The highlight of this study was that older age is more likely to increase fruit, vegetable, and dairy intake.

## 1. Introduction

According to the World Health Organization (WHO), more than 17 million people die annually from cardiovascular diseases (CVDs), making them the leading cause of death worldwide [1]. It is projected that by 2030, the global death toll attributed to CVDs will exceed 23 million, with more than 7 million resulting from complications of coronary artery disease (CAD) [2]. Lifestyle modifications are essential components of the treatment and prevention of CVDs. These modifications include smoking cessation, regular physical activity, and the adoption of healthy eating habits, which are the pillars of these changes [3]. Taking regional aspects into account, cardioprotective dietary patterns share common characteristics. These practices are identified by high amounts of vitamins, minerals, and fiber, along with low concentrations of salt, saturated fats, and lower glycemic indices and loads. Robust studies emphasize the importance of healthy dietary patterns in the prevention of CVDs [4,5].

Several studies show that the adherence to the Mediterranean dietary pattern reduces the risk of cardiovascular events [6,7,8]. The Mediterranean diet score is a rating scale that assesses the adherence to the Mediterranean diet, based on selected food groups in accordance with the Mediterranean pattern. It was initially introduced by Trichopoulou in 1995, and since then, various modifications of the original score have occurred over time [9]. As a disadvantage, these modifications had specific cut-off points for the studied population, making them non-replicable in countries outside of Europe. This limitation restricts the use of the tool, as it cannot facilitate comparisons between different populations [10]. Recently, a study developed a scoring system that uses absolute cut-off values based on a review of the food intake distribution in previous Mediterranean diet score studies. This unifies the tool and allows its application across various populations [11].

In September 2021, an update of the guidelines for the prevention of CVDs was published by the European Society of Cardiology [12]. This clinical practice guide was based on several recommendations for lifestyle changes, including dietary ones. In this context, studies that verify whether individuals who have suffered from acute myocardial infarction (AMI) adhere to nutritional recommendations made by health professionals based on guidelines, as well as those that assess adherence to the Mediterranean diet using a score with absolute cut-off points are still unknown in Brazil.

Regarding the assessments conducted by this study, the 2021 European Society of Cardiology (ESC) guidelines and the Mediterranean diet score complement each other. Analysis using the 2021 ESC guidelines helps understand if the dietary recommended practices are being followed. Meanwhile, using the Mediterranean diet score allows the understanding of the dietary habits of individuals who have recently experienced a heart attack, thereby enabling the creation of new actions that emphasize the need for a cardioprotective diet for these individuals [11,12].

Previously, the Brazilian Cardioprotective Diet (DICA Br) was developed, which is based on feasible prescriptions and guided by nutritional recommendations aimed at improving the dietary adherence of adults with any atherosclerotic cardiovascular disease. Overall, the DICA Br presents easily achievable nutritional recommendations for the Brazilian population. Thus, the composition of this diet is readily accessible, with full utilization of foods, prioritizing regional foods accepted by participants (rice, beans, fruits, vegetables, and soybean oil). However, these recommendations do not exclusively address individuals who have suffered a heart attack. Despite the 2021 ESC guidelines being of European origin, their application in Brazilian regions is interesting, given the scarcity of studies that address Brazil in understanding the dietary habits of individuals who have experienced a heart attack [13].

The objective of this study is to characterize the dietary consumption in terms of macronutrients and energy in a sample of individuals with recent AMI (between 60 and 180 days) and assess their adherence to the Mediterranean diet using a Mediterranean diet score while also examining their adherence to the recommendations of the ESC 2021 guidelines. Our hypothesis is that patients with recent AMI have a dietary pattern that is consistent with the recommendations of the ESC 2021 guidelines.

## 2. Materials and Methods

### 2.1. Study Design and Study Population

This was a cross-sectional study based on information collected at the baseline of the multicenter DICA-NUTS study [14], which aimed to investigate the effects of the Brazilian cardioprotective diet, with or without supplementation with mixed nuts, on the cardiometabolic parameters of individuals with AMI between 60 and 180 days post-event. For the present study, the inclusion and exclusion criteria of the DICA-NUTS study were used. Participants aged ≥ 40 years with ST-segment elevation myocardial infarction (STEMI) or non-ST-segment elevation myocardial infarction (NSTEMI), as defined by guidelines and duly confirmed through medical records, were included. The exclusion criteria were indications for coronary revascularization surgery (graft/bypass); Human Immunodeficiency Virus (HIV) positive undergoing treatment/Acquired Immunodeficiency Syndrome (AIDS); chronic inflammatory diseases; cancer; substance abuse/alcoholism; chronic use of anti-inflammatory drugs, anticonvulsants, and immunosuppressive drugs; pregnancy or lactation; wheelchair users unable to undergo anthropometric evaluation; extreme obesity [body mass index (BMI) ≥ 40 kg/m^2^]; use of dietary supplements; rejection/allergy to nuts; and participation in other randomized studies whose intervention demonstrably interferes with the primary outcome.

The study was conducted from January 2022 to December 2022. The participating centers were as follows: Associação Beneficente Síria (Hcor) in São Paulo, Institute of Cardiology (IC-FUC) in Rio Grande do Sul, Federal University of Goiás (UFG) in Goiás, Federal University of Maranhão (UFMA) in Maranhão, Federal University of Rio Grande do Norte (UFRN) in Rio Grande do Norte, Federal University of Alagoas (UFAL) in Alagoas, Federal University of Paraná (UFPR) in Paraná, hospital of clinics de Porto Alegre (HCPA) in Rio Grande do Sul, and finally, the National Institute of Cardiology (INC) in Rio de Janeiro. A total of 488 individuals were recruited, considering all the centers involved in the DICA-NUTS study.

Individuals were recruited through an analysis of the cardiology service appointment schedules of all the study centers, with a review of medical records to verify the eligibility criteria. Those who agreed to participate in the study were then invited to attend the centers at the scheduled time to begin their participation in the research.

This study was conducted in accordance with the Helsinki Declaration amended in 2013 and was approved by the Scientific Committee/Research Ethics Committee of the National Institute of Cardiology under number 5.412.876 and CAAE: 57427522.1.0000.5272 approved on 17 May 2022. Upon arrival, the participants received explanations and instructions about the research procedures, and the informed consent form was read and signed. After obtaining participant consent, a standardized questionnaire was administered, collecting sociodemographic, clinical, and lifestyle information via an electronic version, registered in the exclusive system of the DICA-NUTS study. Anthropometric assessment and dietary and physical activity inquiry methods were also applied among the participants.

### 2.2. Anthropometric Assessment

Body weight was measured in kilograms (kg) using an electronic anthropometric scale from the brand Líder^®^ (São Paulo, Brazil), with a maximum capacity of 200 kg and accuracy of 100 g, positioned on a flat surface. The participants were weighed barefoot.

Height was measured in meters using a stadiometer (with a 1 mm accuracy), also from the brand Líder^®^, attached to the scale, with individuals barefoot, standing upright with their arms extended along the body. Weight and height measurements were used to calculate the BMI, by dividing the weight (kg) by the height (m) squared and classified according to the guidelines proposed by the WHO for individuals over 18 years old and elderly individuals over 60 years old, according to the data from the Food and Nutrition Surveillance System (SISVAN) [15,16].

Waist circumference (WC) and hip circumference (HC) measurements were taken using a sturdy, non-stretchable, flexible measuring tape with a precision of 0.1 cm. The circumference values were expressed in centimeters (cm) to one decimal place. WC measurement was taken at the midpoint between the lower edge of the ribcage and the iliac crest at the mid-axillary line; HC was defined as the measurement of the widest part of the hip region (with the participant’s legs together at the time of measurement). The circumference data (waist and hip) were used to calculate the waist-to-hip ratio (WHR), classified according to the WHO guidelines.

### 2.3. Food Frequency Questionnaire

A food intake assessment was conducted using the 24 h dietary recall (24DR) and a Food Frequency Questionnaire (FFQ) employing the version used in the ISACAMP study [17]. In the 24DR, the participants reported their food intake over the 24 h prior to the interview. During this process, detailed information was gathered regarding the types of foods consumed, food preparation methods, portion sizes, household measures, quantities, and meal times during the 24 h preceding the consultation. The FFQ was employed to assess the habitual food consumption of the evaluated population. In this context, the questionnaire structure allowed for the recording of the food consumption frequency in units of time (days, weeks, or months).

To aid in the utilization of the 24DR and FFQ, a photographic album with standardized household measures was employed. The interviews were conducted with the aim of obtaining information that would enable the definition and quantification of food intake during the reference period, without inducing responses. For the analysis of the nutritional composition of energy and nutrients from the 24DR data obtained, a computerized system was utilized (Vivanda) [18], which prioritizes Brazilian nutritional composition tables.

### 2.4. Mediterranean Diet Score

The instrument used for collecting data regarding the dietary intake was the FFQ developed and validated for the ISACAMP study, which aims to assess the health profile of the population [17]. In this regard, the structure of the instrument included recording the frequency of food consumption in units of time (days, weeks, or months).

Food consumption data were recorded in Microsoft Excel 2016 spreadsheets. Subsequently, the grams of food groups were calculated using a rule of three, based on the book *Tabela para Avaliação de Consumo Alimentar em Medidas Caseiras*, 5th edition [19]. Then, the grams were recalculated using a rule of three to adjust them to time measures (day and week) for subsequent application of the Mediterranean diet score.

The Mediterranean diet score used in the study was the one proposed by Sofi et al. according to Table 1 [11]. The score consists of 9 components (vegetables, fruits, legumes, fish, meat and meat products, dairy, cereals, alcohol, and olive oil) with a scoring scale from 0 to 18 points. Participant adherence was classified as low (0 to 7 points), moderate (8 to 10 points), and high (11 to 17 points).

### 2.5. European Society of Cardiology ESC Guidelines 2021

On 30 August 2021, the ESC published new guidelines for the primary and secondary prevention of atherosclerotic cardiovascular disease (ASCVD), endorsed by 12 European professional societies. The guidelines discuss recommendations for disease prevention in clinical practice, including individuals who are elderly, with and without ASCVD, with familial hypercholesterolemia, diabetes, and chronic kidney disease [12].

Regarding diet, the guidelines emphasize reducing the consumption of animal-derived foods and promoting the consumption of plant-based foods, given the published edition’s concern for environmental sustainability, supporting this dietary pattern change. As a level A recommendation, the adherence to the Mediterranean dietary pattern for preventing ASCVD is given the top priority. Additionally, reducing the saturated fat intake, with guidance to consume up to 500 g of red meat and its derivatives per week, also aligns with level A recommendations according to the guidelines’ recommendation table [11]. Below is Table 2 with the recommendations of the ESC 2021 guidelines.

Following the respective recommendations outlined in the publication, the guidelines also advise reducing the salt intake and encourages a plant-based dietary pattern, including fruits, vegetables, whole grains, and dietary fibers. Regarding alcohol consumption, there is a limit of up to 100 g per week, and recommendations regarding fish consumption are also provided in the publication. These recommendations are part of the official publication with high levels of importance for ASCVD prevention, providing crucial guidance for healthcare professionals in clinical practice who are involved in managing these individuals [11].

### 2.6. Statistical Analysis

The analyses were performed using Stata Data Analysis and Statistical Software version 15.0. Values were considered statistically significant when the *p*-value < 0.050.

The normality of variable distribution was assessed using the Kolmogorov–Smirnov test. Numeric variables with a normal distribution were described using the mean and standard deviation (SD), while those with a non-normal distribution were described using the median and interquartile range (IQR). Categorical variables were described using the absolute frequency (n) and relative frequency (%).

Raw and adjusted logistic regression analyses (for dichotomous classification variables) and ordinal logistic regression analyses (for variables with three categories) were employed, with the odds ratio (OR) and 95% confidence interval (95% CI) as measures of effect. Variables with a *p*-value < 0.200 in the raw analyses were selected for adjusted models.

The final model was constructed using the backward method, meaning that variables were removed one by one in increasing order of the *p*-value until only those with a *p*-value < 0.050 remained.

## 3. Results

The total sample of participants was 488 individuals, 290 from the South region, 111 from the Northeast region, 71 from the Southeast region, and 16 from the Central–West region.

### Sample Characterization

The characterization of the studied sample is presented in Table 3.

The dietary data on daily consumption of macronutrients and micronutrients in the present study, as shown in Table 4, were obtained through the application of the FFQ.

The mean energy intake of the sample was 1512 kcal (IQR 1161–1985), with 52% from carbohydrates, 18.5% from proteins, and 29.5% from lipids. Saturated fatty acids represented 9.71% of the total intake, monounsaturated fatty acids 8.6%, and polyunsaturated fatty acids 6.3%. The cholesterol intake of the sample was 219.2 mg, with 8.6% from omega-6 and 0.9% from omega-3. The sodium intake was 2674 mg per day.

Applying the Mediterranean diet score to the analyzed sample, as shown in Table 5, we have the median points acquired according to the adherence to the Mediterranean diet. Based on the scoring, the mean points for the fruit group were 1 point, followed by 0 points for vegetables, 2 points for meat, 0 points for fish consumption, 2 points for alcohol consumption, 0 points for the cereal group, 2 points for legume consumption, and 2 points for dairy consumption, totaling 6 points as the median total count.

According to the scores, the analysis of the adherence to the Mediterranean diet using the Mediterranean diet score proposed by Sofi et al. [11], was considered moderate, totaling 9 points.

An analysis of the adequacy of the ESC 2021 guideline recommendations for the secondary prevention of cardiovascular diseases was carried out to assess whether the participants followed the recommendations (Table 6).

According to these analyses, the fruit consumption was in accordance with the recommendation (51.6%), the vegetable intake was below the recommendation at 93.0, the meat consumption was within the recommendations at 64.1%, the fish intake was within the recommendation (91.8%), the alcohol intake was at 85.7%, the sodium intake showed 95.1% adequacy, and the fiber intake was below the recommendation at 79.7%.

The factors associated with an increase in the Mediterranean diet score according to the food groups were analyzed, as shown in Table 7. It is noteworthy that, in the fruit group, being aged ≥ 60 years old (OR = 1.63; 95% CI = 1.18–2.24), never smoking (OR = 2.58; 95% CI = 1.41–4.72), or being a former smoker (OR = 2.17; 95% CI = 1.21–3.88) were factors that remained directly associated in the multiple model with an increase in the fruit intake score.

Regarding the vegetable intake score, being female (OR = 1.68; 95% CI = 1.12–2.52)] and having a higher education (OR = 1.94; 95% CI = 1.33–2.82)] were factors that remained directly associated with an increase in the score. Similarly, in the multiple analysis model, the meat group also had a higher chance of scoring according to the applied score, for females (OR = 1.49; 95% CI = 1.01–2.51), non-white skin color (OR = 2.11; 95% CI = 1.33–3.33)], and never smoking (OR = 2.52; 95% CI = 1.41–5.24).

Fish consumption had a higher chance when the participants had a non-white skin color (OR = 1.82; 95% CI = 1.23–2.70)], had never smoked (OR = 2.71; 95% CI = 1.19–6.13)], or were former smokers (OR = 3.31; 95% CI = 1.54–7.57)]. The chance of scoring in the alcohol group was higher in the participants aged ≥ 60 years old (OR = 2.01; 95% CI = 1.20–3.36)], females (OR = 2.54; 95% CI = 1.31–5.13), and those who had never smoked (OR = 2.82; 95% CI = 1.28–6.21).

Regarding dairy consumption, only those aged ≥ 60 years old had less chances of scoring on the score (OR = 0.62; 95% CI = 0.44–0.98). With respect to cereal consumption, an age ≥ 60 years old was inversely associated with a higher score (OR = 0.22; 95% CI = 0.08–0.60). Regarding legumes, upon analysis, none of the variables were associated with the score.

Also, an analysis was conducted to identify the factors associated with compliance with the ESC 2021 guidelines, as shown in Table 8.

As in the score ratings, the multivariate model also found greater chances of compliance in the fruit group for participants aged ≥ 60 years old (OR = 1.63; 95% CI = 1.44–1.91), those who had never smoked (OR = 1.34; 95% CI = 1.17–1.65), or were former smokers (OR = 1.36; 95% CI = 1.19–1.69).

Regarding vegetable consumption, being female (OR = 1.42; 95% CI = 1.20–1.86) and having a higher education (OR = 1.37; 95% CI = 1.17–1.77) were also factors related to a higher chance of compliance with the consumption of these foods.

Concerning meat consumption, being female (OR = 1.21; 95% CI = 1.05–1.45) and having a non-white skin color (OR = 1.26; 95% CI = 1.06–1.44) were factors that remained directly associated with an improvement in compliance with the ESC 2021 recommendations. Compliance with fish consumption also showed a higher probability. Being single, widowed, or divorced (OR = 1.35; 95% CI = 1.15–1.82) increased the chance of compliance with the guideline recommendations for fish consumption.

Alcohol consumption had a higher chance of compliance when the participants were aged ≥ 60 years old (OR = 1.48; 95% CI = 1.28–1.82) and were female (OR = 1.40; 95% CI = 1.20–1.81). It is worth noting that regarding salt consumption, no statistically significant values and factors associated with a higher score were found.

Being aged ≥ 60 years old (OR = 1.45; 95% CI = 1.29–1.71) and being a former smoker (OR = 2.78; 95% CI = 1.05–7.37) were factors that remained directly associated in the multiple model with improved compliance with the ESC 2021 recommendation regarding fiber.

Univariate and multivariate analyses were performed. According to our work, we placed more emphasis on multivariate analysis because they had statistical significance. The complete table with all analyses is in the Supplementary Material Table S1.

## 4. Discussion

When analyzing the adherence to the Mediterranean diet among the participants at the baseline of the DICA-NUTS study, a moderate adherence to this dietary pattern was found. This may be justified by the regional factors. Brazil is a country not bathed by the Mediterranean Sea and does not follow the same dietary pattern as individuals residing in that region. Despite obtaining the result of moderate adherence according to our assessment via the Mediterranean diet score, our study found a relatively higher adherence compared to previous studies [11,20,21]. For instance, in a study conducted in 2006 by Panagiotakos [21], a Mediterranean diet score was applied aiming to assess the lifestyle habits of coronary artery disease patients. The study found a low adherence to the Mediterranean diet protocols, despite being in a Mediterranean region like Greece, in contrast to our study.

In 2019, an analysis of the dietary lipid profile in patients undergoing elective cardiac surgery was performed by Ferreira et al. [22], where it was demonstrated that the lipid consumption was below the recommended levels, with the saturated fatty acid intake within the recommendations, reaching up to 7% of the total energy value. Consistent with our study, regarding the total fat consumption, the data found by Ferreira et al. [22] are similar. The total fat consumption by the participants showed a median of 29%, with the saturated fatty acid intake slightly above 9%. However, concerning the recommendations, the saturated fatty acid intake in the sample was high. It can be observed that the total fat intake was within the recommended range; however, the emphasis is on the quality of these fats, with the saturated fat consumption being higher than in the previous studies [23,24]. It is important to say that we analyzed the dietary consumption data of the sample through a computerized system called Vivanda [18]. However, it seems that this profile has not changed despite the availability of information and nutritional assistance in major cardiology centers. Relating the recommendations of the ESC guidelines to the participants’ food intake, it is evident that the population meets the recommendations for fruit consumption, a result similar to that found in the PURE study, published in 2017, which involved more than 135,000 participants and found a high fruit consumption [25]. However, vegetable consumption is still below the recommended levels, as well as fish. In 2020, a study conducted with individuals who had suffered acute myocardial infarction, which aimed to analyze the impact of the Mediterranean dietary pattern on the prognosis of these individuals, found a different result from our study with regard to vegetable consumption. This study demonstrated that the sample investigated obtained a satisfactory vegetable intake [26]. The participants’ diet was also low in fiber, with an intake below 30 g, followed by a low cereal consumption, which may explain the low fiber consumption recommended by the ESC 2021 guidelines. The same result was found in a study conducted in 2018 to analyze the eating patterns of heart patients. In this study, a daily fiber intake of less than 25 g was found. These results can be explained by the low intake of whole foods that occurs in these individuals [27].

A notable point in our study is the low alcohol consumption, which falls within the ESC recommendations and Mediterranean diet score as preventive measures for CVDs. The sample that we evaluated had a low dietary intake of dairy products according to the Mediterranean diet score analyses. The meat consumption, specifically beef, dried meat, sun-dried meat, and bacon, was within the guideline recommendation. According to the ESC 2021 guidelines, meat consumption should be a maximum of 350 to 500 g per week. In this sense, the individuals participating in the study presented an adequate consumption compared to the ESC 2021 recommendation. Statistically analyzing variables such as age, gender, skin color, and smoking, they were present, demonstrating a strong odds ratio for scores related to the food groups in the score and compliance with the recommendations of the ESC 2021 guidelines. Age was strongly related to higher scores, supporting historical data that the elderly pay more attention to their health, always being in contact with healthcare teams and being advised about diet, especially in cardiovascular aspects. The female gender was also one of the variables most related to higher scores and adherence to the recommendations, perhaps because women historically are more committed to incorporating new dietary habits and are more easily reachable by healthcare teams. The education level also had the influence of mainly a higher score in relation to vegetable consumption. This can be justified by the information and knowledge about the importance of these foods being present in the diet of individuals who have suffered a heart attack.

It is noticeable that, from a macronutrient perspective, the participants’ diet was satisfactory. However, it is still necessary to reinforce the guidelines highlighting the importance of micronutrients in regard to cardiovascular health. It is known that food choices are based not only on individual preferences but also influenced by economic and social factors. Therefore, it is important to encourage practices and public policies with a better income distribution to enhance the access to food, ensuring the respect, protection, and promotion of the human right to health.

## 5. Conclusions

We observed that the participants in our study who had recently suffered a heart attack showed moderate adherence to a cardioprotective diet. Notably, food groups such as vegetables, cereals, and fish received low scores, indicating inadequacies in the consumption of these foods. Conversely, food groups such as fruits, meats, dairy, and legumes received higher scores, indicating dietary practices more aligned with the Mediterranean pattern. The analysis of these individuals’ diets revealed non-compliance with cardiovascular secondary prevention guidelines, such as those established by the ESC in 2021. Not all the points recommended by the guidelines are followed by these individuals, resulting in observed deficiencies, especially in regard to fiber and vegetable intake. Regarding macronutrients such as carbohydrates, proteins, and lipids, it was observed that they are within the recommendations established by the guidelines. However, we highlight the alterations in the quality of certain nutrients, such as the high consumption of saturated fat and cholesterol. Although carbohydrates and proteins are aligned with the recommendations, addressing these inadequacies in lipid quality is necessary to promote a healthier diet consistent with the cardiovascular prevention guidelines.

## 6. Study Limitations

One limitation which we observed was the questionnaire used to assess the participants’ diet. We know that these tools can overestimate or underestimate the frequency of food intake and the portions consumed by participants. Even though there is extensive training in the use of this tool, it is subject to error. Another important limitation of the study was the collection of data on the consumption of extra virgin olive oil. We know that this food is present in the Mediterranean diet that we assessed in our study. However, the food frequency questionnaire used did not include the item of extra virgin olive oil, thus characterizing a limitation in our analyses. We also understand the regional aspect of food as a limitation of our work, since eating habits change according to the region. Regionality is an aspect that needs to be considered as a limitation.

## Figures and Tables

**Table 1:** Scoring of components in the Mediterranean diet score proposed by Sofi et al., 2014 [11].

Mediterranean Diet Components	Component Scoring Criteria Mediterranean Diet Score		
	0 Points	1 Point	2 Points
Vegetables (g/day)	<100 g <1 serving/day	100–250 g 1 to 2 and a half servings/day	>250 g >2 and a half servings/day
Cereals (g/day)	<130 g <1 serving/day	130–195 g 1 to 1 and a half servings/day	>195 g >1 and a half servings/day
Fruits (g/day)	<150 g <1 serving/day	150–300 g 1 to 1 and a half servings/day	>300 g >2 servings/day
Legumes (g/week)	<70 g <1 serving/week	70–140 g 1 to 2 servings/week	>140 g >2 servings/week
Fish (g/week)	<100 g <1 serving/week	100–250 g 1 to 2 and a half servings/week	>250 g >2 and a half servings/week
Meat and meat products (g/day)	>120 g >1 and a half servings/day	80–120 g 1 to 1 and a half servings/day	<80 g <1 serving/day
Dairy (g/day)	>270 g >1 and a half servings/day	180–270 g 1 to 1 and a half servings/day	<180 g <1 serving/day
Alcohol (g/day)	>24 g >2 servings/day	<12 g <1 serving/day	12–24 g 1 to 2 servings/day

**Table 2:** Nutritional recommendations from the ESC 2021 guidelines.

ESC Guideline Recommendations
Saturated fatty acids should represent <10% of the total energy intake, through replacement with PUFAs, MUFAs, and carbohydrates from whole grains
Consumption of trans fats should be reduced
Salt consumption should be below 5 g per day
Fiber intake between 30 and 40 g per day, preferably whole
Fruit consumption equal to or above 200 g per day
Vegetable consumption equal to or above 200 g per day
Red meat should be reduced to a maximum of 350 to 500 g per week, in particular, the consumption of processed meat should be minimized.
Consumption of fish 1 to 2 times a week
Consumption of 30 g of nuts or chestnuts
Alcohol consumption should be up to 100 g per week
The consumption of sugary drinks should be discouraged

Source: 2021 ESC guidelines [12]. Note: ESC = European Society of Cardiology; PUFAs = polyunsaturated fatty acids; MUFAs = monounsaturated fatty acids.

**Table 3:** Characterization of the study population at baseline, Brazil (n = 488).

Variables	Total n (%)
Age (years) [Mean (±SD)]	59.5 (±9.4)
Sex	
Masculine	352 (72.1)
Feminine	136 (27.9)
Skin color	
White	335 (68.7)
Brown	89 (18.2)
Yellow	8 (1.6)
Black	54 (11.1)
Indigenous	2 (0.4)
Education	
Illiterate/Incomplete Elementary I	101 (20.7)
Fundamental I complete/Fundamental II Incomplete	91 (16.7)
Complete Elementary II/Incomplete Middle School	68 (13.9)
Complete Secondary/Incomplete Higher Education	143 (29.3)
Graduated	85 (17.4)
Marital status	
Married	279 (57.2)
Single	80 (16.4)
Widower	33 (6.8)
Divorced	60 (12.3)
Stable union	36 (7.4)
Smoking	
Non-Smoker	178 (36.5)
Ex-Smoker	259 (53.1)
Smoker	51 (10.4)
STEMI	
No	171 (35.0)
Yes	317 (65.0)
AMI treatment	
Clinical	45 (9.2)
Angioplasty	44 (9.0)
Angioplasty with stent	386 (79.1)
Bypass	13 (2.7)
Previous stroke	
No	461 (94.5)
Yes	27 (5.5)
Previous angina	
No	230 (47.1)
Yes	258 (52.9)
Previous MRI	
No	459 (94.1)
Yes	29 (5.9)
DM 1 preview	
No	482 (98.8)
Yes	6 (1.2)
DM 2 preview	
No	349 (71.5)
Yes	139 (28.5)
SAH preview	
No	171 (35.0)
Yes	317 (65.0)
Previous dyslipidemia	
No	288 (59.0)
Yes	200 (41.0)

Note: N = absolute frequency; % = relative frequency; SD = standard deviation; AMI = acute myocardial infarction; Previous stroke = previous cerebrovascular incident; Previous MRI = myocardial revascularization; DM 1 = type 1 diabetes mellitus; DM 2 = type 2 diabetes mellitus; SAH = systemic arterial hypertension; STEMI = ST-segment elevation myocardial infarction

**Table 4:** Daily macronutrient consumption of the study population (N = 488).

Variables	Median (IQR)
Macro and Micronutrients	
Energy intake, kcal/day	1512 (1161–1985)
Carbohydrates, % of energy	52.0 (45.1–58.7)
Protein intake (g kg BW^−1^ day^−1^)	0.92 (0.16–4.39)
Proteins, % of energy	18.5 (15.0–23.1)
Total fat, % of energy	29.2 (23.3–35.2)
SFAs, % of energy	9.71 (7.3–12.7)
MUFAs, % of energy	8.6 (6.5–11.3)
PUFAs, % of energy	6.3 (4.8–8.5)
n-3 PUFA (mg)	0.9 (0.5–1.3)
n-6 PUFA (mg)	8.6 (5.3–12.8)
Cholesterol (mg)	219.2 (122.5–370.3)
Sodium (mg)	2674 (1877–3655)

Note: IQR = interquartile range; BW = body weight; MUFAs = monounsaturated fatty acids; PUFAs = polyunsaturated fatty acids; SFAs = saturated fatty acids.

**Table 5:** Assessment of the median adherence score to the Mediterranean diet using the Mediterranean diet score (N = 488).

Food Group	Mediterranean Diet Score Median (IQR)
Fruits	1 (0–2)
Vegetables	0 (0–1)
Meat	2 (1–2)
Fish	0 (0–1)
Alcohol	2 (2–2)
Cereals	0 (0–1)
Legumes	2 (2–2)
Dairy	2 (0–2)
Total	6 (5–8)

Note: IQR = interquartile range.

**Table 6:** Prevalence of individuals in relation to compliance with the ESC 2021 recommendation (N = 488).

Food Group	ESC 2021	n (%)
Fruits	Adequate	252 (51.6)
	Inappropriate	236 (48.4)
Vegetables	Adequate	34 (7.0)
	Inappropriate	454 (93.0)
Meat	Adequate	313 (64.1)
	Above	175 (35.9)
Fish	Adequate	448 (91.8)
	Inappropriate	40 (8.2)
Alcohol	Adequate	418 (85.7)
	Inappropriate	70 (14.3)
Salt	Adequate	464 (95.1)
	Inappropriate	24 (4.9)
Fibers	Below recommendation	389 (79.7)
	Adequate	61 (12.5)
	Above	38 (7.8)

Note: ESC = European Society of Cardiology; N = absolute frequency; % = relative frequency.

**Table 7:** Factors associated with increased Mediterranean diet score.

Variables	Fruits ^a^	Vegetables ^a^	Meat ^a^	Fish ^a^	Legumes ^a^	Alcohol ^a^	Dairy ^a^	Cereals ^a^	Total Adequacy ^b^
	Multi ^c^	Multi ^d^	Multi ^e^	Multi ^f^	Multi	Multi ^g^	Multi ^h^	Multi ^i^	Multi ^i^
	OR (CI 95%)	OR (CI 95%)	OR (CI 95%)	OR (CI 95%)	OR (CI 95%)	OR (CI 95%)	OR (CI 95%)	OR (CI 95%)	OR (CI 95%)
Age (years)									
<60	1.00	-	-	-	-	1.00	1.00	1.00	-
≥60	1.63 (1.18–2.24) *	-	-	-	-	2.01 (1.20–3.36) *	0.62 (0.44–0.98) *	0.22 (0.08–0.60) *	-
Sex									
Masculine	-	1.00	1.00	-	-	1.00	-	-	-
Feminine	-	1.68 (1.12–2.52) *	1.49 (1.01–2.51) *	-	-	2.54 (1.31–5.13) *	-	-	-
Education									
Until incomplete secondary education	-	1.00	-	-	-	-	-	-	-
From full medium onwards	-	1.94 (1.33–2.82) *	-	-	-	-	-	-	-
Marital status									
Married or stable union	-	-	-	-	-	-	-	-	-
Single, widowed, or divorced	-	-	-	-	-	-	-	-	-
Skin Color									
White	-	-	1.00	1.00	-	-	-	-	1.00
Not white	-	-	2.11 (1.33–3.33) *	1.82 (1.23–2.70) *	-	-	-	-	1.70 (1.13–2.57) *
Smoke									
Never smoked	2.58 (1.41–4.72) *	-	2.52 (1.41–5.24) *	2.71 (1.19–6.13) *	-	2.82 (1.28–6.21) *	-	-	-
Ex-smoker	2.17 (1.21–3.88) *	-	1.29 (0.71–2.35)	3.31 (1.54–7.57) *	-	1.90 (0.92–3.91)	-	-	-
Smoker	1.00	-	1.00	1.00	-	1.00	-	-	-
Physical activity levels									
Low	-	-	-	-	-	-	-	-	-
Moderate	-	-	-	-	-	-	-	-	-
High	-	-	-	-	-	-	-	-	-

Note: OR = odds ratio; CI = confidence interval; Multi = multivariate; ^a^ ordinal logistic regression; ^b^ logistical regression. The following were selected for the multiple model: ^c^ age, marital status, and smoking; ^d^ gender, education, and level of physical activity; ^e^ sex, race, and smoking; ^f^ age, sex, marital status, race, and smoking; ^g^ age, sex, education, and smoking; ^h^ age and skin color; ^i^ skin color and smoking. * *p*-value < 0.050.

**Table 8:** Factors associated with compliance with the ESC 2021 recommendation.

Variables	Fruits ^a^	Vegetables	Meat ^b^	Fish ^a^	Alcohol ^a^	Salt ^a^	Fibers ^b^
	Multi ^c^	Multi ^d^	Multi ^e^	Multi ^f^	Multi ^g^	Multi ^h^	Multi ^i^
	OR (CI 95%)	OR (CI 95%)	OR (CI 95%)	OR (CI 95%)	OR (CI 95%)	OR (CI 95%)	OR (CI 95%)
Age (years)							
<60	1.00	-	-	-	1.00	-	1.00
≥60	1.63 (1.44–1.91) *	-	-	-	1.48 (1.28–1.82) *	-	1.45 (1.29–1.71) *
Sex							
Masculine	-	1.00	1.00	-	1.00	-	-
Feminine	-	1.42 (1.20–1.86) *	1.21 (1.05–1.45) *	-	1.40 (1.20–1.81) *	-	-
Education							
Until incomplete secondary education	-	1.00	-	-	-	-	-
From full medium onwards	-	1.37 (1.17–1.77) *	-	-	-	-	-
marital status							
Married or stable union	-	-	-	1.00	-	-	-
Single, widowed or divorced	-	-	-	1.35 (1.15–1.82) *	-	-	-
Skin Color							
White	-	-	1.00	1.00	-	-	-
Not white	-	-	1.23 (1.06–1.44) *	0.38 (0.18–0.62) *	-	-	-
Smoke							
Never smoked	1.34 (1.17–1.65) *	-	-	-	-	-	2.04 (0.75–5.56)
Ex-smoker	1.36 (1.19–1.69) *	-	-	-	-	-	2.78 (1.05–7.37) *
Smoker	1.00	-	-	-	-	-	1.00
Physical activity levels							
Low	-	-	-	-	-	-	-
Moderate	-	-	-	-	-	-	-
High	-	-	-	-	-	-	-

Note: OR = odds ratio; CI = confidence interval; Multi = multivariate; ^a^ logistical regression; ^b^ ordinal logistic regression. The following were selected for the multiple model: ^c^ age, marital status, and smoking; ^d^ gender, education, and physical activity levels; ^e^ gender, marital status, and skin color; ^f^ age, marital status, and skin color; ^g^ age, sex, and education; ^h^ no variables; ^i^ age and smoking. * *p*-value < 0.050; *p*-value < 0.001.

## Data Availability

The dataset used in this study is available from the corresponding author upon reasonable request.

## Supplementary Materials

The following supporting information can be downloaded at: https://www.mdpi.com/article/10.3390/life15071051/s1, Table S1: Factors associated with increased Mediterranean diet score.
