# Supplementary material for: Evaluating Food Intake of Post-Acute Myocardial Infarction Patients According to a European Guideline and Mediterranean Diet Score: DICA-NUTS Substudy
Source: Life (Basel). 2025 Jun 30;15(7):1051. doi: 10.3390/life15071051 (PMC12298716; doi:10.3390/life15071051)
Supplement: Supplementary file 1 [file life-15-01051-s001.zip › life-3522779-supplementary.pdf]

Supplemental data

Table S1. Factors associated with increased Mediterranean diet score.

Variables	Fruits[a]		Vegetables[a]		Meat[a]		Fish[a]		Legumes [a]	
	Uni	Multi[c]	Uni	Multi[d]	Uni	Multi[e]	Uni	Multi[f]	Uni	Multi
	OR (IC 95%)	OR (IC 95%)	OR (IC 95%)	OR (IC 95%)	OR (IC 95%)	OR (IC 95%)	OR (IC 95%)	OR (IC 95%)	OR (IC 95%)	OR (IC 95%)
**Age (years)**										
<60	1.00	1.00	1.00	-	1.00	-	1.00	-	1.00	-
≥60	1.63 (1.17-2.26)*	1.63 (1.18-2.24)*	1.22 (0.84-1.17)	-	1.22 (0.83-1.78)	-	0.72 (0.50-1.04)	-	1.20 (0.71-2.02)	-
**Sex**										
Masculine	1.00	-	1.00	1.00	1.00	1.00	1.00	-	1.00	-
Feminine	1.06 (0.74-1.52)	-	1.58 (1.06-2.36)*	1.68 (1.12-2.52)*	1.50 (0.96-2.33)	1.49 (1.01-2.51)*	0.69 (0.45-1.05)	-	1.20 (0.66-2.19)	-
**Education**										
Until incomplete secondary education	1.00	-	1.00	1.00	1.00	-	1.00	-	1.00	-
From full medium onwards	0.93 (0.67-1.29)	-	1.86 (1.28-2.69)*	1.94 (1.33-2.82)*	0.99 (0.67-1.45)	-	1.18 (0.82-1.72)	-	0.84 (0.50-1.41)	-
**Marital status**										
Married or stable union	1.00	-	1.00	-	1.00	-	1.00	-	1.00	-
Single, widowed or divorced	0.72 (0.51-1.01)	-	0.93 (0.63-1.37)	-	1.27 (0.85-1.91)	-	0.70 (0.47-1.04)	-	1.05 (0.60-1.81)	-
**Skin Color**										

White	1.00	-	1.00	-	1.00	1.00	1.00	1.00	1.00	-
Not white	0.98 (0.69-1.40)	-	0.71 (0.47-1.07)	-	2.01 (1.28-3.15)*	2.11 (1.33-3.33)*	1.80 (1.21-2.66)*	1.82 (1.23-2.70)*	1.24 (0.69-2.21)	-
**Smoke**										
Never smoked	2.54 (1.39-4.63)*	2.58 (1.41-4.72)*	1.39 (0.71-2.72)	-	2.52 (1.32-4.82)*	2.52 (1.41-5.24)*	2.66 (1.18-5.99)*	2.71 (1.19-6.13)*	1.78 (0.75-4.18)	-
Ex smoker	2.19 (1.23-3.92)*	2.17 (1.21-3.88)*	1.30 (0.67-2.49)	-	1.19 (0.66-2.14)	1.29 (0.71-2.35)	3.33 (1.51-7.35)*	3.31 (1.54-7.57)*	1.42 (0.64-3.15)	-
Smoker	1.00	1.00	1.00	-	1.00	1.00	1.00	1.00	1.00	-
**Physical activity levels**										
Low	1.00	-	1.00	-	1.00	-	1.00	-	1.00	-
Moderate	1.20 (0.82-1.76)	-	1.46 (0.94-2.24)	-	0.83 (0.53-1.30)	-	0.94 (0.62-1.44)	-	0.96 (0.52-1.76)	-
High	1.33 (0.85-2.07)	-	1.43 (0.86-2.39)	-	0.77 (0.46-1.31)	-	0.98 (0.59-1.62)	-	0.83 (0.41-1.69)	-

Table S1. Factors associated with increased Mediterranean diet score (continue).

Variables	Alcohol[a]		Dairy[a]		Cereals[a]		Total Adequacy [b]	
	Uni	Multi[g]	Uni	Multi[h]	Uni	Multi[i]	Uni	Multi[i]
	OR (IC 95%)	OR (IC 95%)	OR (IC 95%)	OR (IC 95%)	OR (IC 95%)	OR (IC 95%)	OR (IC 95%)	OR (IC 95%)
**Age (years)**								
<60	1.00	1.00	1.00	1.00	1.00	1.00	1.00	-
≥60	2.01 (1.21-3.32)*	2.01 (1.20-3.36)*	0.62 (0.44-0.98)*	0.62 (0.44-0.98)*	0.22 (0.08-0.60)*	0.22 (0.08-0.60)*	1.18 (0.79-1.75)	-
**Sex**								
Masculine	1.00	1.00	1.00	-	1.00	-	1.00	-
Feminine	2.55 (1.30-5.00)*	2.54 (1.31-5.13)*	0.94 (0.63-1.39)	-	0.33 (0.10-1.14)	-	1.19 (0.77-1.84)	-
**Education**								
Until incomplete secondary education	1.00	-	1.00	-	1.00	-	1.00	-
From full medium onwards	1.06 (0.63-1.78)	-	1.07 (0.75-1.54)	-	0.62 (0.27-1.43)	-	1.25 (0.84-1.86)	-
**Marital status**								
Married or stable union	1.00	-	1.00	-	1.00	-	1.00	-
Single, widowed or divorced	0.88 (0.52-1.49)	-	1.19 (0.82-1.73)	-	0.86 (0.36-2.04)	-	0.88 (0.58-1.33)	-
**Skin color**								
White	1.00	-	1.00	-	1.00	-	1.00	1.00
Not white	0.98 (0.69-1.40)	-	1.53 (1.03-2.27)*	-	1.75 (0.78-3.96)	-	1.70 (1.13-2.57)*	1.70 (1.13-2.57)*
**Smoke**								

Never smoked	2.46 (1.41-5.32)*	2.82 (1.28-6.21)*	0.66 (0.34-1.30)	-	0.61 (0.16-2.57)	-	1.89 (0.91-3.96)	-
Ex smoker	1.71 (0.84-3.46)	1.90 (0.92-3.91)	0.48 (0.25-1.25)	-	0.98 (0.27-3.51)	-	1.24 (0.60-2.56)	-
Smoker	1.00	1.00	1.00	-	1.00	-	1.00	-
**Physical activity levels**								
Low	1.00	-	1.00	-	1.00	-	1.00	-
Moderate	1.21 (0.69-2.01)	-	0.91 (0.61-1.37)	-	1.85 (0.70-4.88)	-	1.27 (0.81-2.02)	-
High	1.27 (0.64-2.52)	-	0.89 (0.54-1.44)	-	0.99 (0.27-3.58)	-	1.19 (0.68-2.06)	-

Note: OR= odds ratio; CI= confidence interval; Uni= univariate; Multi= multivariate; [a]logistical regression; ; [b]ordinal logistic regression. The following were selected for the multiple model: [c]age, marital status and smoking; [d]gender, education and physical activity levels; [e]gender, marital status and skin color; [f]age, marital status and skin color; [g]age, sex and education; [h]no variables; [i]age and smoking. *p-value<0.050; £p-value<0.001
